# Unified Representation of Twitter and Online News Using Graph and Entities

**DOI:** 10.3389/fdata.2021.699070

**Published:** 2021-08-27

**Authors:** Munira Syed, Daheng Wang, Meng Jiang, Oliver Conway, Vishal Juneja, Sriram Subramanian, Nitesh V. Chawla

**Affiliations:** ^1^ University of Notre Dame, Notre Dame, IN, United States; ^2^ Conde Nast, New York, NY, United States

**Keywords:** twitter, social media, named entities, graph embedding, online news consumption

## Abstract

To improve consumer engagement and satisfaction, online news services employ strategies for personalizing and recommending articles to their users based on their interests. In addition to news agencies’ own digital platforms, they also leverage social media to reach out to a broad user base. These engagement efforts are often disconnected with each other, but present a compelling opportunity to incorporate engagement data from social media to inform their digital news platform and vice-versa, leading to a more personalized experience for users. While this idea seems intuitive, there are several challenges due to the disparate nature of the two sources. In this paper, we propose a model to build a generalized graph of news articles and tweets that can be used for different downstream tasks such as identifying sentiment, trending topics, and misinformation, as well as sharing relevant articles on social media in a timely fashion. We evaluate our framework on a downstream task of identifying related pairs of news articles and tweets with promising results. The content unification problem addressed by our model is not unique to the domain of news, and thus can be applicable to other problems linking different content platforms.

## 1 Introduction

One of the goals of online news providers is to improve customer satisfaction by recommending relevant articles in a timely fashion. To personalize content recommendations, news providers may collect user attributes such as demographic information or assess user interests through their chosen articles. However, when a user accesses a certain service for the first time, it is difficult to ascertain their interests. This is the cold start problem in recommender systems and recent works have been leveraging social media to address it (Lee et al. (2014); Hsieh et al. (2016)). Using social media for news personalization and recommendation has already shown promise in several works (Trevisiol et al. (2014); Lin et al. (2014); Ashraf et al. (2018)).

While news outlets were a dominant mode of news consumption for most people, recently social media has become a popular source of news information. Not only do people share news articles on social media, thus giving us insight into their topic interests, but they also discuss these topics through posts, comments, and reactions, providing insight into their sentiment and opinions. Thus, by combining news and social media data, there is now an opportunity to incorporate users’ interests and their opinions on various topics in personalization and recommendation models. We pose the following questions in this paper: How to integrate the news and social media posts to develop a more complete engagement profile of consumers? Can social media inform news consumption patterns and can news consumption patterns inform social media activity?

This task of unifying news and social media, however, is not trivial due to the differences in expression used in the two including formality, slang, memes, emoticons, length of text, and different intentions in communication. News outlets generally aim to inform and are not as biased as social media posts, which may be posted to convince others to adopt a particular opinion or express an opinion or sentiment on a topic. Despite this bias, in cases like hazard detection, political events, or crowd-sourced applications, social media platforms such as Twitter provide a gold mine of information that news may not be able to capture. This interplay between news and social media also gives us an opportunity to study how social media affects journalism.

Besides differences in how language is used, various technical challenges exist in accomplishing this task. In particular, Twitter imposes a limit on the number of characters that users are allowed to post. Thus, the context available to glean the topic of the tweet is quite limited compared to news articles. Apart from that, the use of different words to refer to the same concept or entity poses a challenge in inferring the topic of the tweet. On the other hand, while individual tweets are usually focused on one topic, each news article may cover multiple topics. Therefore, it is harder to detect the central topic to the article which should be used to find the relevant tweet.

To overcome these challenges, in this paper we propose a model to build a unified representation of both types of content by using a graph of news articles and tweets. This generalized graph representation can be used for different downstream tasks such as identifying sentiment, trending topics, and misinformation, as well as finding relevant news articles to share on social media in a timely manner. We focus on using an entity-based framework to connect tweets to news articles. Recently, the use of entities has become popular for linking disparate sources of information. For example, Spitz and Gertz (2018) use an entity-centric framework to detect new events and track them across multiple news sources. We then build a tripartite graph of news articles, entities, and tweets using various NLP techniques that would represent the unified content space. We evaluate our unified representation on the task of identifying tweets that are relevant to news articles. We show the effectiveness of this approach through different experiments and evaluation measures.

### 1.1 Related Work

The increasing use and prevalence of social media has not only changed how people communicate with each other, but also intertwined it with other media and types of content consumption such as news (Bruns and Burgess (2012); Vis (2013)). This prevalence has also lead to citizen journalism (Bruns et al. (2012)) and ambient journalism (Hermida (2010)) through social media. Furthermore, news organizations have also started using these social media platforms to promote their content and engage their users (Hermida et al. (2012)). On the flip side, some studies have also reported on the use of social media for journalistic purposes such as news reporting (Broersma and Graham (2012); Paulussen and Harder (2014); Priya et al. (2019)). In some cases, breaking news was first reported on social media like Twitter before mainstream news media had covered it (Hu et al. (2012); Vis (2013)).

The advent of the Internet has also encouraged user-interaction and user-generated content in association with online news. Studies have found that incorporating user comments through forums improve the recommender systems (Li et al. (2010); Bach et al. (2016)). Trevisiol et al. (2014) found that incorporating the browsing behavior of users through referrer URLs improved the recommendation of articles by building a *BrowseGraph* and *ReferrerGraph*. Other studies have found a more direct connection between user content and news. For example, Tatar et al. (2014) used the comments posted by users to rank news articles and infer their popularity, and Kourogi et al. (2015) proposed a model that suggests attractive news headlines to share on social media.

The idea of using social media for online news personalization and recommendation is an established one and motivated by past research. Lin et al. (2014) treat the opinions of social media influencers as auxiliary information in their news recommendation model and demonstrate the effectiveness of this method on the cold-start problem. Recently, recommendation frameworks also include social media preferences of users (Ashraf et al. (2018)) and make news recommendations using users’ social media information when available. Given such intermingling of news and social media, it is worthwhile to explore a unified view of the two content spaces. Another benefit of creating such a representation is that tweets are generally short and lack context. Thus, news articles can provide the required context to support NLP tasks on tweets such as topic modeling and opinion mining (Guo et al. (2013)).

Some studies have proposed a unified framework to represent multiple news channels. For example, Mele et al. (2019) use a topic modeling and Hidden Markov Model based approach for event detection and tracking through different news streams. Spitz and Gertz (2018) use named entities to aggregate news from multiple streams. They also use a graph to represent all the content to support further downstream analysis. Similar to them, we use an entity-based framework due to the different style of languages used in news articles and tweets.

The problem of linking news to tweets has been tackled in other studies. Guo et al. (2013) use hashtags, named entities, or temporal constraints with a latent variable model Weighted Textual Matrix Factorization to link news with tweets. They use the title and a summary to represent the news article. Suarez et al. (2018) use Named Entity Recognition and article text summarization in their methods for linking them. Wang et al. (2015) also propose a unified framework to find the most relevant news articles to a particular tweet by mining multi-aspect reflections. Another interesting and related problem tackled by Wei and Gao (2015) is using tweets to summarize news articles. They find relevant tweets that share links to the news articles and use the text of the tweets as reference summaries for training their supervised learning model for news text summarization. The problem of generating relevant summarized social media discussion has also been tackled by Chakraborty et al. (2017) wherein they use a network based unsupervised approach to handle the noise and diversity of tweets. Li et al. (2016) describe EKNOT, their framework that summarizes events using both news and social media perspectives. Their system presents a higher level summary and overview of the events, while our framework attempts to unify the content representation at a granular level.

While Twitter has been linked with news for NLP tasks, it is also useful for answering questions about journalism and the relationship of news with social media. Wihbey et al. (2017) use Twitter to understand the relationship between journalists and social media. Tsagkias et al. (2011) consider the task of finding republished articles on social media in the domain of online reputation management, where organizations monitor their online reputation by leveraging social media. Republished articles could also generate new discussions around the topic. One of the applications of building a unified graph representation is to monitor the discussions surrounding news articles. Holton et al. (2013) study the motivation of Twitter users behind linking news articles on Twitter. Hong (2012) shows that the adoption of social media improves the online readership of newspapers. Kumar et al. (2017) predict which news articles will generate discussion on social media based on their content. Morgan et al. (2013) explore the relationship between the perceived ideology of news outlets and the sharing of news on social media. Lehmann et al. (2013) detect related discussion of tweeters after they tweet a particular news article. Bruns and Burgess (2012) discuss some approaches that can be used to link news to Twitter discussions with the help of keywords and hashtags, identifying temporal patterns and key users, and using graphs for analysis. We aim to support analyses such as these and future work in this area through the unified content representation of the two spaces generated by our framework.

In the rest of this section, we provide the background on relevant topics.

### 1.2 Named Entity Recognition

Since our framework utilizes entity-based techniques, in the next few paragraphs we provide an overview of existing techniques. Named Entity Recognition (NER) is defined as the task of extracting names of entities such as names of people, organizations, and locations from text (Yadav and Bethard (2019)). This task generally consists of two steps: 1) the demarcation of the string in the text that is identified as an entity and 2) annotating the entity with its type, such as organization, person, location, and time (Basile and Caputo (2017)). Recently, NER methods have started using deep-learning algorithms instead of feature-engineering based techniques. Yadav and Bethard (2019) show that neural networks that infer features perform better than feature-engineering systems. NER methods such as a NER tagger provided by Stanford NLP toolkit (Manning et al. (2014)), models that use LSTMs (Lample et al. (2016); Chiu and Nichols (2016)) and conditional random fields (McCallum and Li (2003); Settles (2004)) are just a few of many NER models that have been proposed over the years.

Many works focus on identifying named entities in social media. In microblogs such as Twitter, the challenge of identifying entities is exacerbated due to noise, informal language, grammatical errors, a lack of capitalization, and spelling errors as well as a lack of sufficient context due to short message lengths (Li et al. (2012); Limsopatham and Collier (2016)). Limsopatham and Collier (2016) use a bidirectional-LSTM and word embeddings to learn entities from tweets. Li et al. (2012) propose a framework for identifying named entities in Twitter using both the local context of the tweet as well as global context through Wikipedia. In our framework, we also use both these contexts in identifying and linking the named entities. Efforts have also been made in entity annotation of the Twitter corpus (Derczynski et al. (2016)) by humans, a relatively more expensive undertaking compared to unsupervised models. The task of entity recognition has useful applications to governments and companies such as hazard detection and early crisis response (Li et al. (2012)). Further, the quality of entities detected can be improved by linking them to a knowledge base. For example, Yamada et al. (2015) link the entities to Wikipedia to improve the identification of entities in Twitter.

### 1.3 Named Entity Linking

Since we wish to connect tweets to news articles by linking entities, the text from each source needs to be linked to the correct entities. This brings us to the problem of disambiguation and aliasing. Entity disambiguation refers to the task of linking entities when multiple of them share the same name but refer to different entities. This commonly occurs when multiple people share the same name. For example, a poet and an Olympic gold medalist share the same name Kevin Young, but are different people. However, when an article refers to the poet, it should be connected to a separate entity than when it links to the athlete. The Wikipedia disambiguation page for Kevin Young shows seven different people at the time of this writing (“Kevin Young”). Another related problem is aliasing, in which a particular entity could be referenced in multiple ways. A common example of this is a person’s name, which could be written in different formats including the first name and last name, initials only, last name only, etc. An example to the Wikidata article on Donald Trump shows 14 aliases in English alone (“Donald Trump”). In general, the steps for entity linking are as follows:

1) Use a Named Entity Recognition system to identify entities in a text,
2) Generate a set of candidate entities using a knowledge base such as Wikipedia,
3) Rank the candidate entities using their prior probability and the context of the text in which the entity is present,
4) Select the most likely entity from the candidate set as the linked entity.

Given that tweets not only use different ways of referring to an entity, but also include additional complications due to non-standard language and spelling errors, many works have explored the problem of entity linking in Twitter. Basile and Caputo (2017) provide an overview of entity linking methods to be used specifically for tweets. Urata and Maeda (2017) use Wikipedia for word-sense disambiguation of entities in tweets. Waitelonis and Sack (2016) use a DBPedia knowledge base to link entities in tweets. Thus, we also link entities in our framework to knowledge bases, so that we can take advantage of the context and prior probability for entity disambiguation and aliasing.

### 1.4 Coreference Resolution

While these methods aim to learn entities from tweets instead of general news domain, our problem requires us to be able to learn entities from both, the news and Twitter. Most tweets have only one or two entities, since each tweet is focused on one topic usually. However, identifying entities from news provides the opposite challenge. News articles tend to have many entities including locations, dates, and times along with persons and organizations. However, for the task of connecting these news articles with tweets many of these entities are irrelevant to the tweet and we need to find the important ones.

One way of dealing with this problem is using coreference resolution. Coreference resolution is the task of finding all the references in a text made to a particular entity occurring elsewhere in that text. For example, pronouns typically refer to some entity in the sentence. The task of coreference resolution is to identify which pronouns are related to which entities in the sentence and cluster them correctly. Modern methods rely on deep neural networks as they perform better than syntactic parsers and feature-engineering based methods (Gu et al. (2018)). The various coreference resolution models can be broadly categorized into mention pair classifiers, entity-level models, latent-tree models, mention-ranking models, and span-ranking models (Gu et al. (2018); Lee et al. (2017)). The models proposed by Clark and Manning (2016a,b) are examples of a mention-ranking models. The models by Lee et al. (2017) and Gu et al. (2018) are examples of span-ranking models. In our framework we use a mention-ranking model that is described in further detail in Section 2.2.3.

### 1.5 Graph Embeddings

Graph embeddings have become a popular way of lower dimensional representation of vertices in the graph. Modern embedding algorithms tend to fall in the categories of matrix factorization, random walking, and graph neural networks (Cui et al. (2018)). In our analysis, we use a random walking based method. These methods are based on the word2vec model by Mikolov et al. (2013), which uses a skipgram or continuous bag of words architecture to learn embeddings of words using the neighborhood of words in a sentence to train the neural network. Random walk based algorithms such as deepwalk (Perozzi et al. (2014)), node2vec (Grover and Leskovec (2016)), and metapath2vec (Dong et al. (2017)) use a random walking scheme to express the graph nodes as words in a sentence. The steps are as follows:

1) Random walking to generate sequence of nodes,
2) Use a skipgram architecture with one hidden layer that will be used to infer the embeddings of nodes.

We use these embeddings to identify similar nodes in our unified graph.

## 2 Materials and Methods

### 2.1 Data Collection and Preprocessing

In this section, we describe the process of acquiring data used in the construction of the twitter-news graph.

#### 2.1.1 Twitter Data Collection

The online news magazine whose articles we attempt to link to tweets has its own Twitter handle and often tweets its articles. This gives us a good starting point for identifying tweeters who are engaged with the news magazine’s content. By analyzing the content that these users generate, we hope to gain a better understanding of how potential users and subscribers engage with the news content and what their interests are. This is similar to the strategy used by Nigam et al. (2016). Since we are specifically looking for tweets relevant to the news articles, we not only collected tweets containing keywords including the name of the magazine but also streamed tweets of users who were more engaged with tweets generated by the magazine’s twitter handle. By streaming the data, we hope to capture a more complete and representative version of Twitter with respect to news. Thus, the steps we used for collecting tweets are as follows:

1) Collect the most recent tweets by the news magazine’s twitter handle from December 12, 2019 to January 3, 2020. This resulted in 765 tweets.
2) Sample a subset of 2,257 users that retweet the magazine’s tweets. Since the magazine has many followers and likes on each tweet, we selected tweeters through retweets with the expectation that these users are more engaged with the news magazine than followers and users who like the tweets.
3) Stream tweets from December 12, 2019 to January 3, 2020, based on the following criteria. Stream 1,928,699 tweets generated by users selected in step 2 and 765 tweets by the magazine. We also collected tweets containing keywords such as the magazine’s name and various author names who frequently write for the magazine resulting in a total of 3,006,233 collected.

#### 2.1.2 News Data Collection

Since we aim to align news articles with tweets, we collected the content of articles from an online news magazine that were clicked on during the same time period that the tweets were collected. This data was directly obtained from the clickstream log of users who accessed news articles within the time frame that the tweets were collected.

#### 2.1.3 Text Preprocessing

We used the same data preprocessing steps to normalize tweets and news articles. Normalizing tweets is not a trivial task due to the informality of language used including the use of slang, emoticons, and spelling errors, and many research efforts have been made to improve this process. However, this is an important step in our framework because news articles use more formal language. With our aim to unify the text of tweets and news articles, we need to apply certain preprocessing steps such that the informality of Twitter text is reduced. User names, URLs, numerical values including date and time, and email addresses were replaced with a placeholder such as <email> or <url>. Contractions such as “can’t” were expanded, hashtags were separated, and emoticons were replaced. We used *ekphrasis* (Baziotis et al. (2017)) for this data processing step, as they provide a comprehensive library for cleaning the text data and are geared towards text from social media. The following are the steps we used for preprocessing and tokenizing the data:

1) Filter out tweets/articles that are not in English. Since the entities we use are from English corpii, this is an important filtering step for gleaning context.
2) Eliminate duplicate tweets/articles in the corpus. We represent this original corpus of unique texts as *T*_*org*−*twitter*_ and *T*_*org*−*news*_ for tweets and news articles, respectively.
3) Normalize “url”, “email”, “percent”, “money”, “phone”, “user”, “time”, “date”, “number”.
4) Annotate hashtags, elongated words, emphasized words, and censored words in the text.
5) Unpack hashtags such that each word in a hashtag is a separate token. Expand contractions in the text.
6) Using a dictionary, identify and replace emoticons with words in the dictionary. We denote these preprocessed lists of tokens as *T*_*norm*−*twitter*_ and *T*_*norm*−*news*_ for tweets and news articles, respectively.

We use these preprocessed lists of tokens for content representations. However, to identify entities in the text, we use raw data, as punctuation and capitalization are essential components for identifying named entities.

#### 2.1.4 URL Preprocessing

To establish direct connections between news articles and tweets, we parse URLs in all the text. Since URLs can be shortened or have aliases, we first parse the URLs to generate the full-URL. These preprocessed URLs are used for creating connections between tweets and news articles. For this paper, we only consider links mentioned on tweets that are direct URLs of the news articles.

### 2.2 Model

In this section, we explain the various components of the unified graph model. We will also explain the motivation behind various design choices.

#### 2.2.1 Named Entity Representation

Each text document in the corpus is represented as a list of named entities that appear in the tweet or news article. We run the Named Entity Recognition (NER) parser through *T*_*org*−*twitter*_ and *T*_*org*−*news*_. We use spacy’s NER tool (Honnibal and Montani (2017)) to extract entities. Spacy’s Named Entity Recognizer uses a convolutional neural network (Partalidou et al. (2019)) and is trained on OntoNotes 5 corpus and recognizes 18 entity types. However, we only track entities of types PERSON, NORP, FAC, ORG, GPE, LOC, PRODUCT, EVENT, WORK_OF_ART, LAW, and LANGUAGE. We do not include numerical entities including date and time since they generally do not provide useful links between two documents. For example, if one text mentions “two” apples, and another tweet mentions “two” cycles, connecting these texts by the word “two” leads to noisy edges in the graph.

#### 2.2.2 Linked Knowledge Base

While entities were able to provide us with useful edges between text documents, two entities with the same name do not necessarily refer to the same entity. For example, different people with the same name could be mentioned in different text documents, but they would end up getting mapped to the same entity. On the other hand, the same entity could have multiple representations in the corpus. We tackle this problem by using Named Entity Linking and Disambiguation. Entities are linked to a knowledge base, Wikipedia and Wikidata, in our case. Spacy (Honnibal and Montani (2017)) has provided a fast implementation.^1^ of entity linking with the following steps:

1) Input NER mention from the text and generate candidate entities for each mention from the knowledge base.
2) From text, embed the sentence context *s*_*i*_ and entity type of the mention *t*_*i*_.
3) For each candidate entity from the knowledge base, calculate the prior probability *p*_*i*_ and encode the entity description *d*_*i*_.
4) Concatenate *s*_*i*_, *t*_*i*_, *p*_*i*_, and *d*_*i*_ into a single vector and learn the probability of the entity given the mention.

**FIGURE 1:**
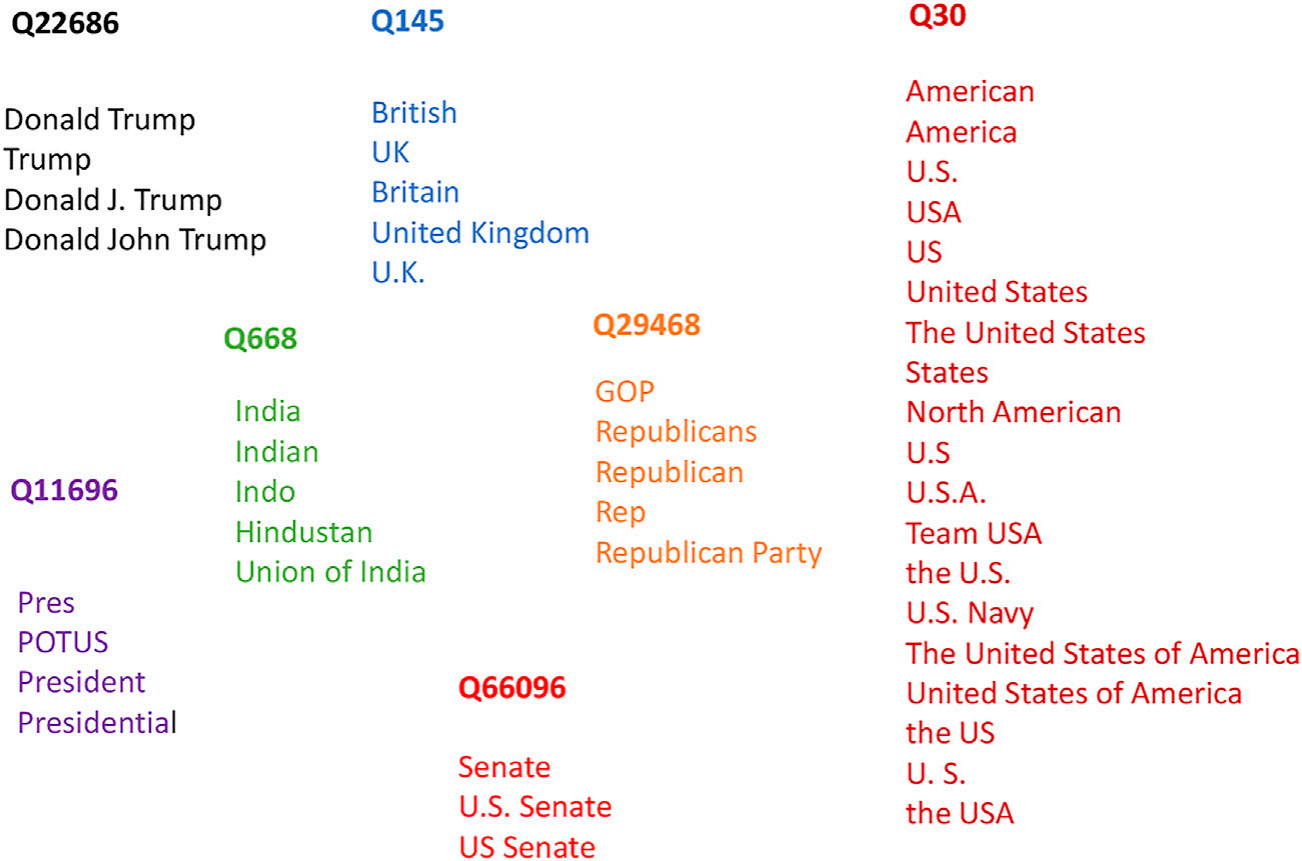
Examples of linked entities.

Figure 1 shows examples of some of the most frequent entities that were aliased in the tweets. We found different variations of the name Donald Trump, country names such as the United States, United Kingdom, and India, and political terms such as President and GOP.

#### 2.2.3 Coreference Resolution

For coreference resolution, we use the implementation provided by Hugging Face.^2^. Their implementation is based on the mention-ranking model by Clark and Manning (2016a), Clark and Manning (2016b). Mention-ranking models use the likelihood of coreference to score pairs of mentions (Clark and Manning (2016a)). The steps for mention-ranking model are:

1) Extract mentions from the text,
2) Compute a set of features for each pair of mentions,
3) Using the features, find the most likely antecedent for each mention. Return clusters of mentions. In Clark and Manning (2016b), they use a learning-to-search to train a neural network to merge clusters, whereas Clark and Manning (2016a) uses a reinforcement learning algorithm to optimize the model for coreference evaluation metrics.

**FIGURE 2:**
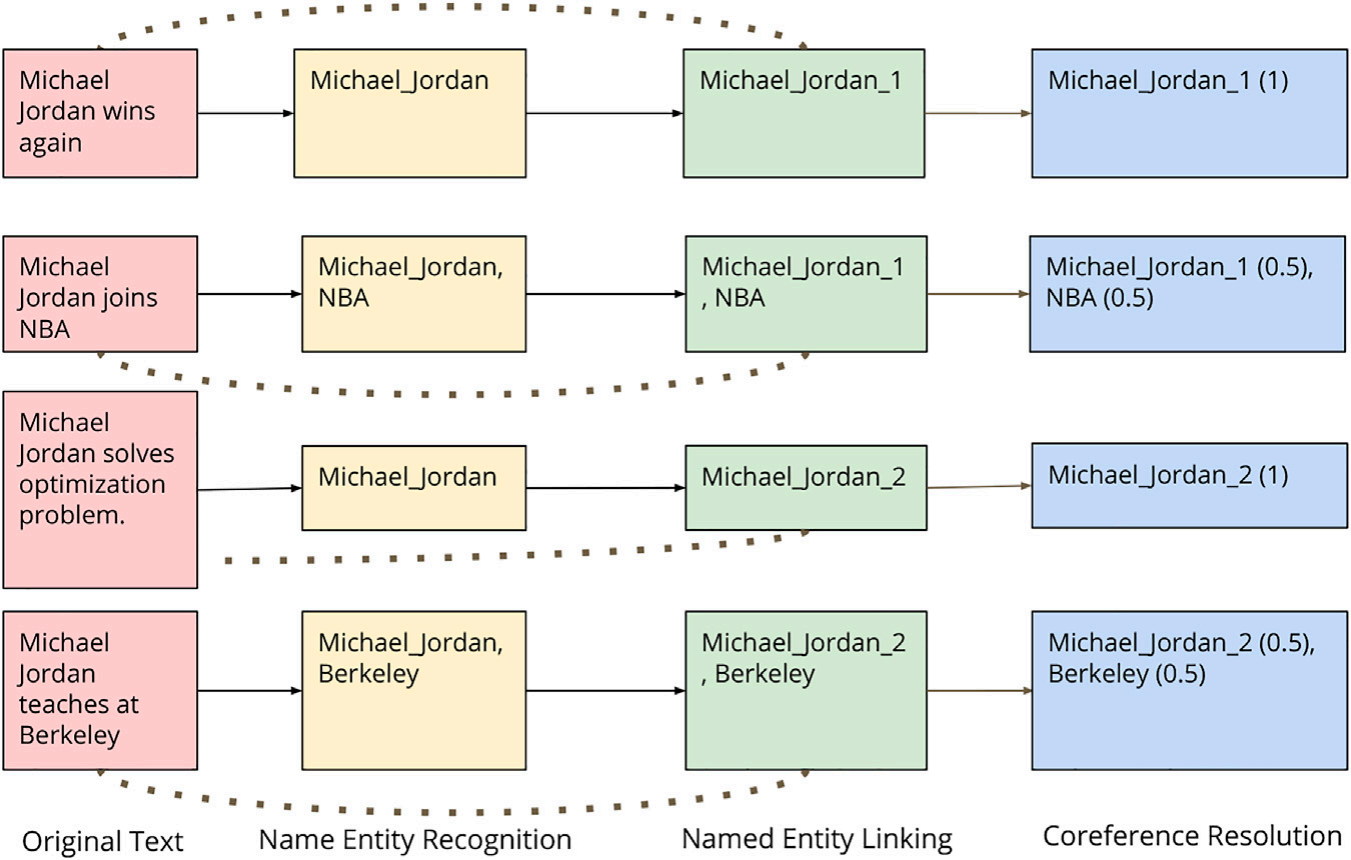
Illustration of different stages.

Figure 2 shows an illustration of various stages on some sample text.

#### 2.2.4 Graph Construction

**FIGURE 3:**
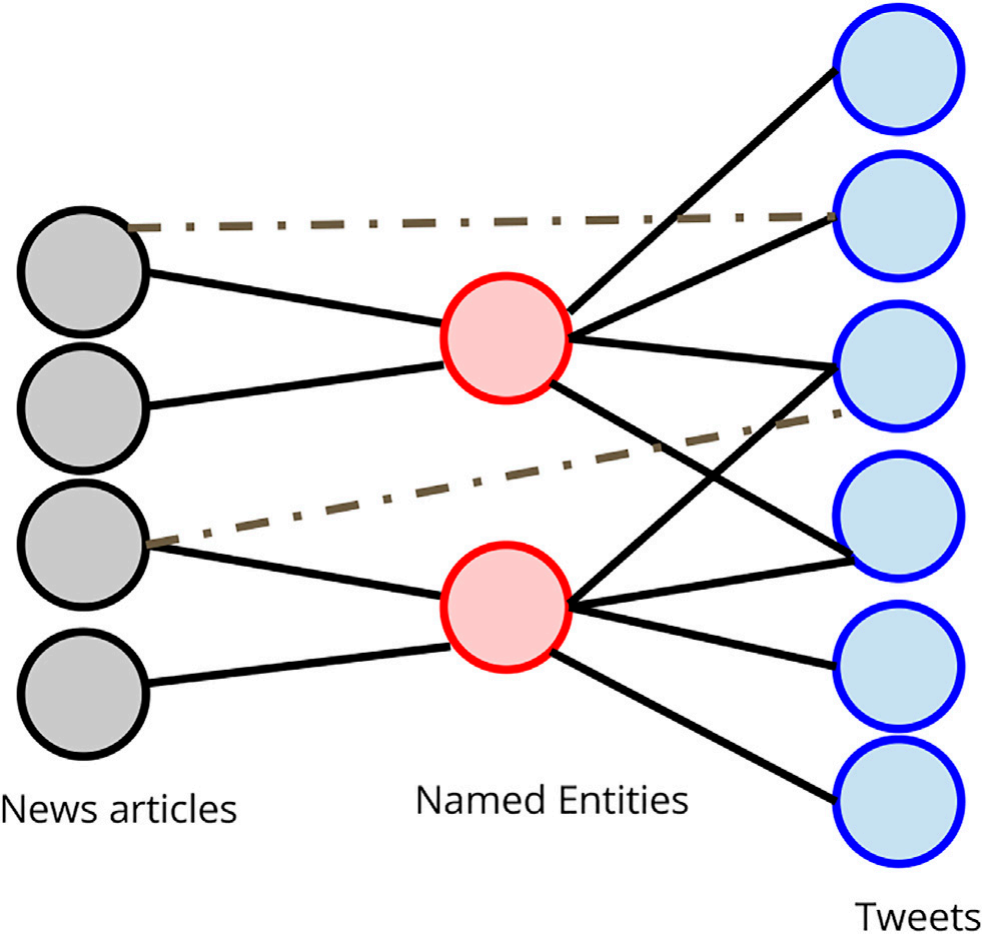
Tripartite graph schema.

We construct a tripartite graph with the first layer being news articles, the second layer being named entities, and the third layer consists of tweets. We restrict the entities to the top 1,000 most frequent entities among the tweets. The set of edges are drawn between tweets and entities *E*_*T*−*E*_ based on whether the tweet contains the entity. The set of edges drawn between the entities and news articles are denoted as *E*_*N*−*E*_ and drawn in a similar fashion. The set of edges between news articles and tweets *E*_*N*−*T*_ are drawn if the tweet directly links the news article in it. These edges are the rarest in the graph. Figure 3 shows a diagram of such a graph. The set of edges *E*_*T*−*E*_ and *E*_*N*−*E*_ are weighted using coreference resolution. The edge is weighted by the size of each coreference cluster in the text. All the *E*_*N*−*T*_ edges have a weight of 1.

### 2.3 Data Description

From all the tweets, we extracted 1,964,367 entities with 68,621 unique entities. Of all the entities extracted, 55,376 occurred in both tweets and news data. Building a tripartite graph with all of these entities led to 460,485 connected components in the graph, with the largest connected component having 392,450 nodes and 710,427 edges. Thus, we have a sparsely connected graph. The number of direct references made from tweet to news article, i.e., *E*_*N*−*T*_ is 53, a low number compared to the other types of edges in the graph. In the tripartite graph, we only keep the most frequent 1,000 entities and retain 21,518 news articles and 369,880 tweets. Including more entities bloats the graph further while making it sparser due to being mentioned less frequently.

**FIGURE 4:**
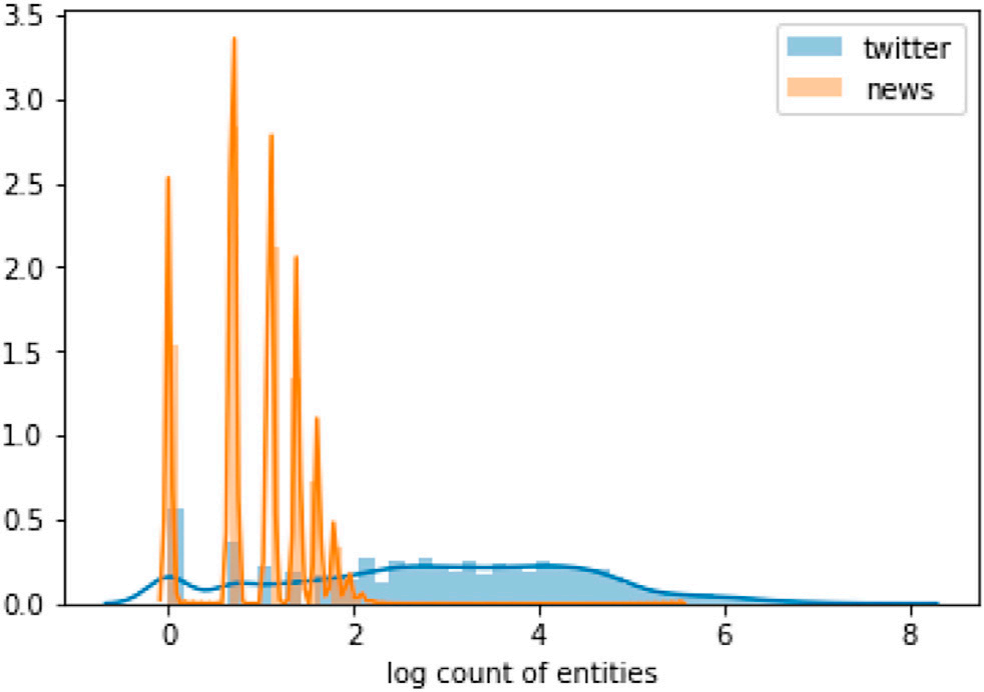
Normalized histogram of the log count of entities in tweets vs. news articles.

Figure 4 shows a normalized histogram of the log count of the number of entities in each document (tweet or article). We see that news articles have more entities in them compared to tweets. In fact, tweets have 1.9 ± 2.0 average entities in a tweet, with the median being 2 entities per tweet. In contrast, news articles have 57.0 ± 117.6 entities on average, with the median being 19 entities per article. In the tripartite graph, of the three groups of nodes, the entities have the highest degree distribution, followed by the news nodes, and then tweets. The degree distribution of the nodes also follows a power-law distribution, with the maximum degree being 49,853, but the mean is 3.6 ± 120.0, and the median is 1. Thus, while the degree of entity nodes can be extremely high, many tweets are only connected to one entity.

## 3 Results

### 3.1 Article-Tweet Relatedness

The annotation of semantic relatedness was one of the tasks in the SemEval-14 challenge (Marelli et al. (2014)), wherein participants submitted a system that could rate the relatedness of two sentences. We propose a task similar to this for evaluation, where we evaluate our framework on the task of finding tweets most similar to news articles and rating their relatedness. We generate embeddings for all the nodes in the constructed graph using a simple random walker and skipgram architecture with negative sampling, the same as node2vec (Grover and Leskovec (2016)) with *p* and *q* set to 1. Then for each news article, we find the *k* most similar tweets by calculating the cosine similarity between each pair of news articles and tweets embeddings. We rank the top 100 news articles with the highest similarity to tweets in the automatic evaluation. For each news article, we report the top *k* most similar tweets.

### 3.2 Evaluation

Since the data was collected from Twitter and News directly, we do not have a ground truth dataset of linked tweets and news articles. To acquire such a ground truth dataset, we would need to know which tweets are related to which news articles beforehand, which is infeasible on Twitter. Annotating linked pairs of news and tweets is tedious and expensive, given the sheer number of tweets we collected. Therefore, we are unable to use the more traditional retrieval metrics of precision at *k* or recall at *k* without a labeled dataset. To calculate these metrics, we would need to label the relatedness of every pair of news article and tweet.

Instead, we consider other methods of evaluation, using both automatic and human evaluation methods as described below. Human evaluation is important to get the subjective perspective of text relatedness. We ask humans to annotate the relatedness of a subset of articles linked by the framework, which provides us the ground truth for evaluation. However, it is expensive, so we also use automatic evaluation methods based on n-gram matching.

We also established a random baseline to compare the performance of our model. As part of this baseline, we randomly match tweets to news articles. This baseline is expected to give us an idea of the volume and diversity of topics in tweets. Since many of the tweets are related to politics, the relatedness of a tweet and news article may not be perfectly random.

#### 3.2.1 Amazon Mechanical Turk

We use Amazon Mechanical Turk to rate the relatedness of a tweet and a news article. Workers were asked to rate whether a tweet and a summarized news article were related or relevant to each other. An example of a related summarized news articles and tweet pair is shown below:

News article: Jana Prikryl Reads Anne Carson: Jana Prikryl joins Paul Muldoon to read and discuss Anne Carson’s “Stanzas, Sexes, Seductions,” and her own poem “Thirty Thousand Islands.”

Tweet: @VChangPoet I keep mentioning this every time someone asks about an amazing book, but I’ll do it again: Anne Carson... https://t.co/XHWQXfCsaW.

We showed workers two sets of news article-tweet pairs. The first set of pairs shown to workers was created using our model. We used cosine similarity on the graph embeddings of each news and tweet to identify the related pairs. The second set is constructed using the random baseline, in which the news articles were paired with random tweets. The annotators could select out of three options: 1). the news article and tweet are completely unrelated, 2). the news article and tweet are broadly related, and more context is needed, and 3). the news article and tweet are definitely related. The news article was represented as the title, followed by a summary of the article with at most 50 words. The summary was generated using TextRank, an algorithm that ranks sentences using the PageRank algorithm. Each pair was assigned three unique mturk workers. We annotated the 30 most similar with 5 tweets each, thus resulting in 150 unique pairs.

Table 1 shows the results of evaluating the 150 pairs generated by the full framework and random baseline. The mean and median rating of the full framework are higher than the random baseline, which is expected. However, we see that the random baseline also has related texts and that a random sampling of the article-tweet pairs are not completely unrelated. This further emphasizes the difficulty of finding relevant pairs in an open dataset where the total number of related pairs is unknown. The workers took a median time of 28 s to finish their tasks.

**TABLE 1:** Amazon mechanical turk evaluation.

Model	Mean	SD	Median
Full Framework	1.91	0.72	2
Random Baseline	1.44	0.73	1

#### 3.2.2 Automatic Evaluation

For automatic evaluation we consider different strategies based on n-grams. Scores such as BLEU and ROUGE are n-gram based evaluation measures that can be used for the task of text summarization, in which a long text is summarized to a shorter version with fewer sentences. These evaluation measures require a gold standard reference summaries provided by humans with which to compare the target summary. Due to the lack of reference summaries, we adopt a different strategy. We treat the tweets as summaries of the news articles to which they are related. Thus, we use the sentences of the news article as references for the related tweets. In other words, if one of the tweets that a news article was paired with was a sentence from the article, the BLEU and ROUGE score would be the highest. While we do not expect high BLEU and ROUGE scores, we expect there to be at least a few n-grams in common which can be matched and use these measures to compare the different methods. We rank the top-10 most related tweets to the news article. Table 2 reports the ROUGE F1-scores of different methods.

**TABLE 2:** Automatic evaluation of text relatedness.

Model	BLEU	ROUGE-1	ROUGE-2	ROUGE-3	ROUGE-4	ROUGE-L	ROUGE-W
Full framework	0.0112	0.0682	0.0075	0.0013	0.0003	0.0907	0.0354
Full framework - coref weighting	0.0056	0.0717	0.0084	0.0016	0.0003	0.0939	0.0356
Full framework using Stanford NER	0.0073	0.0573	0.0017	2.88E-05	0	0.0785	0.0300
Full framework - KB linking	0.0073	0.0682	0.0073	0.002	0.00098	0.09133	0.0355
Random baseline	0.0025	0.0405	0.001	3.08E-05	2.02E-07	0.058	0.0221

##### 3.2.2.1 Bilingual Evaluation Understudy

BLEU (Papineni et al. (2002)) is a precision-related measure (Lin (2004)). While it was originally proposed for the machine translation task, it has also been used for text summarization (Indu and Kavitha (2016)). To calculate the BLEU score, n-gram matches are determined between the reference and target sentences. Then a modified precision score for the entire corpus is calculated by adding the clipped n-gram counts for the reference sentences and dividing by the number of reference n-grams (Papineni et al. (2002)). BLEU scores have a range of 0–1, with 1 being achieved when the tweet perfectly matches one of the sentences in the news article. A score of 1 is very unlikely even in human translated text. A score of 0 means that the texts completely mismatch.

##### 3.2.2.2 ROUGE-N

ROUGE-N recall typically measures the n-gram recall between the target summary and reference summaries generated by humans. In our case, ROUGE-N recall calculates the ratio of overlapping n-grams found in the news article, i.e., the reference, and the corresponding tweet it is paired with by the model to the number of n-grams in the sentences of the news article. ROUGE-N precision calculates the ratio of n-grams overlapping between the target and reference to the number of n-grams in the reference. ROUGE-N F1-score is the geometric mean of ROUGE-N recall and ROUGE-N precision. ROUGE-1 counts unigrams, ROUGE-2 counts bigrams, ROUGE-3 counts trigrams, and ROUGE-4 counts 4-g that overlap in the news article and tweets. As *n* increases, the recall would naturally reduce because overlapping 4-g would be less common than overlapping trigrams, bigrams, and unigrams. We can observe this in Table 2.

##### 3.2.2.3 ROUGE-L

ROUGE-L measures the longest common subsequence between the target and reference texts. ROUGE-L eliminates the problem of choosing a particular *n* for comparison, which is what we do when using ROUGE-N. ROUGE-L simply measures the longest subsequence whether it is unigram, bigram, trigram, or even longer. The longer the longest common subsequence is, the more similar the target document would be to the reference. Another advantage of ROUGE-L is that it does not require consecutive matches as long as the matches occur in the correct sequence within any part of the sentence (Lin (2004)). Thus, we see that ROUGE-L scores are higher than ROUGE-N scores in Table 2.

##### 3.2.2.4 ROUGE-W

ROUGE-W measures the weighted longest common subsequence between the target and reference texts. This is similar to ROUGE-L, except that it weights consecutive matches higher than non-consecutive ones. The weight factor used is 1.2, which is the weight used in the official evaluation package v1.2.1 (Lin (2004)).

We see from the tables that the full model performs the best in BLEU and second-best in ROUGE. The model also performs better than the random baseline. In addition, we also evaluated the performance through ablation studies by removing different components of the model. We also include a comparison with a version of the model that uses StanfordNER (Finkel et al. (2005)) for entities.

##### 3.2.2.5 Ablation Studies

Since our framework includes multiple steps, we can get a better understanding of the performance of it by comparing the changes in performance when the different steps are removed or replaced. Table 2 the performance of the different model modifications across the performance measures defined above.

*Full framework:* This model has all the steps as described in Section 2.2, comprising of Named Entity Representation, Linked Knowledge Base, Coreference Resolution, and Graph Construction. Table 2 shows that the full framework performs the best in BLEU.

*Full framework - coref weighting:* This model includes Named Entity Representation, Linked Knowledge Base, and Graph Construction. The unweighted graph is used for generating embeddings. This model is slightly better than the full framework across ROUGE-1, ROUGE-2, ROUGE-4, ROUGE-L, and ROUGE-W, but its BLEU score is half of the BLEU score of the full model. It is the best performing model when considering the metric of ROUGE-2.

*Full framework using Stanford NER:* In this model, Stanford’s Named Entity Recognition (Finkel et al. (2005)) has been used for identifying named entities instead of Spacy’s NER. From Table 2, we see that the performance of the model drops in both BLEU and all the ROUGE scores compared to the model using spacy’s NER tool in this dataset.

*Full framework - KB Linking:* In this modification, the named entity linking step is removed. The steps included in this model are Named Entity Representation, Coreference Resolution, and Graph Construction. Since there is no entity disambiguation or linking, in the coreference resolution step, all entities with the same name are considered the same. Similarly, the same entity with different names will not be clustered together during coreference resolution. This leads to a drop in the BLEU and ROUGE-2 score, however, this particular model is the best performing of all considering ROUGE-3, ROUGE-4, ROUGE-L, and ROUGE-W metrics.

*Random Baseline:* The random baseline is the same as the one used in Section 3.2.1 for human annotation. Random tweets were paired with the news articles. We see from Table 2 that the random baseline is the worst performing across all the metrics except ROUGE-3 and ROUGE-4.

## 4 Discussion

In this paper, we addressed the problem of unifying content spaces from different platforms, namely news and Twitter, by proposing an entity-based graph representation. We used different NLP techniques in the graph construction process, including named entity recognition and linking and neural coreference resolution. We evaluated the graph on one downstream application, in which we retrieved the most related tweets to a particular news article. We showed that the framework returned more relevant tweets than the baselines both using Amazon Mechanical Turk and automatic evaluation measures of BLEU and ROUGE. Through Amazon Mechanical Turk experiments, we see that the pairs identified by our model are broadly related in topic. Through the random baseline we see that most tweets and news articles are unrelated to each other in topic, which shows the difficulty of the task. Identifying related pairs of tweets and news articles from openly collected datasets is challenging because there is no guarantee that even one pair of the tweets and articles actually matches outside the articles whose links were directly tweeted. However, we were able to discover some related pairs, which should encourage the unification of both these sources of news. While these results are promising, this approach holds even further potential for improvement. In the Amazon Mechanical Turk evaluation, we would have expected all the randomly assigned pairs to be marked completely unrelated, but this was not the case. As many of the news articles and tweets are related to politics, these results are not surprising. Since even the news articles were summarized for the annotators, annotating the relatedness of short texts is challenging due to a lack of context. The difference between broadly related and definitely related texts would be harder to discern when comparing short texts.

We hope to address certain limitations of this proposed framework in the future. In our graph construction process, we did not link between tweets using retweet information and other twitter metadata, but that could potentially add more signal in the graph, thus improving our model. Due to the lack of context, we are unable to leverage more sophisticated contextual embedding algorithms such as ELMO (Peters et al. (2018)). However, by aggregating tweets through clustering or combining retweets, we may be able to leverage more contextual information in the graph construction process, such as weighting edges.

Another direction of exploration we would like to consider in the future is evaluating on more downstream applications. We evaluated our method on one task of text relatedness, through which we were able to successfully retrieve many relevant tweets. However, there is still an error component. To support downstream applications such as sentiment analysis and opinion mining, we would need to reduce the error rate even further. We would also like to answer research questions related to journalism and user interests that this graph would be able to support.

The content unification problem addressed by our model is not unique to the domain of news, and thus can be applicable to other problems linking different content platforms. For example, a unified representation of academic papers and social media would help give insights into scientific outreach and how public interest influences scientific progress. Given the promise showed in this work, our framework has the potential to benefit many downstream applications that require the unification of content across platforms.

## Data Availability

The data analyzed in this study is subject to the following licenses/restrictions: The Twitter data is publicly available via Twitter API. The news content can be accessed through Conde Nast’s website. The clickstream data is not publicly available. Requests to access these datasets should be directed to NC, nchawla@nd.edu.

## Footnotes

1
https://github.com/svlandeg/spaCy/tree/3fbab231b530e6b638c3443cf37c38c62d0e4647/bin/wiki_entity_linking

2
https://github.com/huggingface/neuralcoref
